# A literature-derived benchmarking database for single-ancestry polygenic risk score construction methods with spectral ranking inferences

**DOI:** 10.1093/bib/bbag598

**Published:** 2026-10-30

**Authors:** Chris Sebastian, Mengxin Yu, Jin Jin

**Affiliations:** Department of Mathematics, University of Pennsylvania, 209 South 33rd Street, Philadelphia, PA 19104-6395, United States; Department of Biostatistics, Epidemiology and Informatics, Perelman School of Medicine, University of Pennsylvania, 3600 Civic Center Boulevard, Philadelphia, PA 19104-6021, United States; Department of Statistics and Data Science, Washington University in St. Louis, 1 Brookings Drive, St. Louis, MO 63130, United States; Department of Biostatistics, Epidemiology and Informatics, Perelman School of Medicine, University of Pennsylvania, 3600 Civic Center Boulevard, Philadelphia, PA 19104-6021, United States

**Keywords:** polygenic risk score (PRS), spectral ranking inference, meta-aggregation of literature data, method benchmarking database, uncertainty quantification

## Abstract

Polygenic risk scores (PRSs) have emerged as a valuable tool for genetic risk prediction and stratification in human diseases. Over the past decade, extensive methodological efforts have focused on improving the predictive power of PRS, leading to the development of numerous methods for PRS construction. Benchmarking these various methods thus becomes an essential task that is crucial for guiding future PRS applications. While studies have benchmarked subsets of these methods on specific phenotypes and cohorts, the resulting evidence remains fragmented, with a lack of work that comprehensively assess the relative performance of the various PRS methods. We addressed this gap by constructing a PRS method benchmarking database, synthesizing published results in 2009–2025 and conducting spectral ranking inference with uncertainty quantification to rank 14 single ancestry summary statistics-based PRS methods that had sufficient benchmarking evidence to support reliable ranking. We constructed rankings using two complementary sources: original method-development studies and applications/benchmarking studies. While the highest-ranked methods (LDpred2 and AnnoPred) and the lowest-ranked method (C + T) were consistently identified from both sources, the relative ordering of most methods showed moderate variability. We further constructed phenotype-specific rankings, providing more detailed insights into the robustness and phenotype-specific strengths of individual methods. Collectively, the overall and phenotype-specific rankings of the PRS methods, along with the detailed model performance data curated from the literature, form a dynamic benchmarking database that can be continuously updated as new PRS methods and published benchmarking evidence emerge, providing a practical resource for future PRS method selection and application.

## Introduction

Polygenic risk scores (PRSs), derived from genome-wide association studies (GWAS), are increasingly recognized as quantitative measures of genetic liability with great potential for facilitating disease risk stratification, personalized risk management, and clinical decision-making [1–3]. Recent studies have highlighted the clinical utility of PRS when integrated with complementary data modalities, such as electronic health records [4–7] and multi-omic profiles [8–10]. A wide range of methods have been proposed for PRS construction, with many approaches focusing on the use of GWAS summary-level association statistics, which are far more widely available than individual-level GWAS data. Early approaches, such as LD Clumping and *p*-value Thresholding (C + T) [11, 12]—also known as P + T—and related implementations such as PRSice [13], were widely adopted due to their computational efficiency, algorithmic simplicity, and ease of interpretation. More advanced methods were subsequently developed, including Bayesian approaches (e.g. LDpred2 [14], PRS-CS [15], and SBayesR [16]) and penalized regression-based methods (e.g. lassosum2 [17]). By explicitly accounting for complex correlation structures among millions of genetic variants, these methods have demonstrated improved predictive performance over earlier, model-free approaches.

To date, over 40 methods have been proposed for PRS model development, with individual method-development papers often claiming superiority over existing approaches for specific phenotypes or data scenarios. The relative performance of PRS methods is known to depend on multiple factors, including how well the distribution assumption approximates the true effect-size distribution of genetic variants of a phenotype, algorithm robustness, and other factors [3]. In addition to the original method papers, which typically benchmark newly proposed methods against a subset of existing methods on a limited set of phenotypes for illustrative purpose, subsequent application and benchmarking studies have evaluated different subsets of PRS methods across a broader range of phenotypes and datasets. Together, results reported in both method-development and applied benchmarking papers serve as valuable but fragmented resources for understanding PRS method performance and informing method selection in future applications. However, there remains a lack of synthesis efforts that comprehensively summarize the relative performance of PRS methods across diverse phenotypic categories and data settings. Integrating findings from the existing literature using appropriate analytical frameworks could provide a more systematic assessment of method robustness and phenotype-specific performance patterns. Moreover, a stratified investigation based on the type of paper: either the original method papers or the independent applied and benchmarking studies may further offer a balanced evaluation and provide additional insights.

In this study, we constructed a literature-evidence-based PRS method benchmarking database, consisting of curated benchmarking data extracted from the literature, including detailed model performance metrics such as prediction *R*^2^ and area under the ROC curve (AUC) across studies, and PRS method rankings derived from this data using a spectral ranking inference approach with uncertainty quantification. We identified 14 single-ancestry methods for GWAS summary data-based PRS construction that had been sufficiently compared against each other on complex human phenotypes to support stable ranking inference. Methods developed for multi-ancestry, admixed population, cross-trait, or other settings, as well as newer single-ancestry methods with insufficient comparative evidence, were not included because the available literature did not provide enough connected comparison data for reliable ranking. Method comparison results for these 14 methods were collected from 26 peer-reviewed manuscripts published between 2009 and 2025. These literature-derived data posed a series of challenges for synthesis analysis, including sparsity of pairwise method comparisons and incomparability of results across applications due to differences in evaluation metrics, target phenotypes, and study cohorts. To address these challenges, we adopted a recently proposed spectral ranking inference approach that enables statistically principled aggregation of sparse and heterogeneous comparison data while providing confidence intervals (CIs) for inferred ranks of the PRS methods. Our analysis identified several methods that were consistently ranked at the top (LDpred2 and AnnoPred) or bottom (LDpred2-inf and C + T) with high confidence, based on evidence drawn either from original method-development papers or from subsequent application and benchmarking papers. Meanwhile, the remaining methods had rankings with largely overlapping CIs, suggesting similar overall performance. From the method paper-based rankings, we observed two clusters of methods where one (including LDpred2, AnnoPred, LDpred2-auto, LDpred-funct, and DBSLMM) outperformed the other (including PRS-CS, SCT, LDpred, PRS-CS-auto, LDpred2-inf, and C + T). We also observed a notable pattern that the rankings tended to be higher for methods proposed more recently. On the other hand, with a larger number of applications across a wider range of phenotypes, the rankings based on application papers gave much narrower CIs for most methods and the methods’ ranks were less related to their publication dates. While most methods’ ranks were comparable between rankings derived from method-development studies and those based on applied or benchmarking studies, a small number of methods (including SCT and DBSLMM) showed notable differences in rank between the two rankings. We further conducted phenotype-specific summary analyses, generating rankings stratified by phenotype. These analyses revealed substantial variability in methods’ relative performance across phenotypes and provided more detailed insights into the robustness and phenotype-specific strengths of individual PRS methods. Together, the overall and phenotype-specific ranking results, along with the curated literature-based benchmarking data, provide a comprehensive reference database for benchmarking PRS methods and guiding method selection in future PRS development and applications across diverse complex human phenotypes.

## Materials and methods

### Problem setup and data preparation

We constructed a literature data-based PRS method benchmarking reference database. The database consists of two complementary components: (1) curated reported benchmarking results extracted from the literature in the form of prediction performance metrics, such as *R*^2^ and AUC values (Supplementary Table S1); and (2) comprehensive PRS method performance rankings with uncertainty quantification, including overall ranking, ranking stratified by publication source, and phenotype-specific ranking, that were derived from the curated empirical evidence. Specifically, we first identified 14 methods for GWAS summary data-based PRS construction that had accumulated sufficient head-to-head comparisons with each other with real data evidence from diverse studies across multiple categories of phenotypes to support reliable and stable ranking inference. These methods include C + T [12, 18], LDpred [11], lassosum [19], AnnoPred [20], PRS-CS-auto [15], PRS-CS [15], SBayesR [16], SCT [21], DBSLMM [22], LDpred2-inf [14], LDpred2-auto [14], LDpred2 [14], LDpred2-funct [23], and lassosum2 [17], sorted by publication date (from earliest to latest). We observed that these eligible methods selected were all commonly implemented single-ancestry, summary data-based methods in the standard PRS construction setting where a single GWAS summary dataset is available for model training, referred to as the single ancestry setting. Other categories of PRS methods, such as those in the multi ancestry setting where ancestry-stratified GWAS summary data are available from more than one ancestry population for model training, those in the admixed population setting aiming to improve PRS performance on admixed populations, and cross-trait methods aiming to improve PRS performance by borrowing information across related traits, have mostly not been sufficiently compared in the literature and are thus not included in our analysis. Finally, we collected 26 papers from peer-reviewed manuscripts published between 2009 and 2025 that reported method comparison results among the 14 methods based on 531 applications across 114 distinct phenotypes and 6 superpopulations (Fig. 1). These phenotypes span across several categories of human complex traits including binary clinical endpoints and disease outcomes, continuous physiological traits (e.g. blood lipids, hematological traits), and behavioral traits.

**Figure 1:**
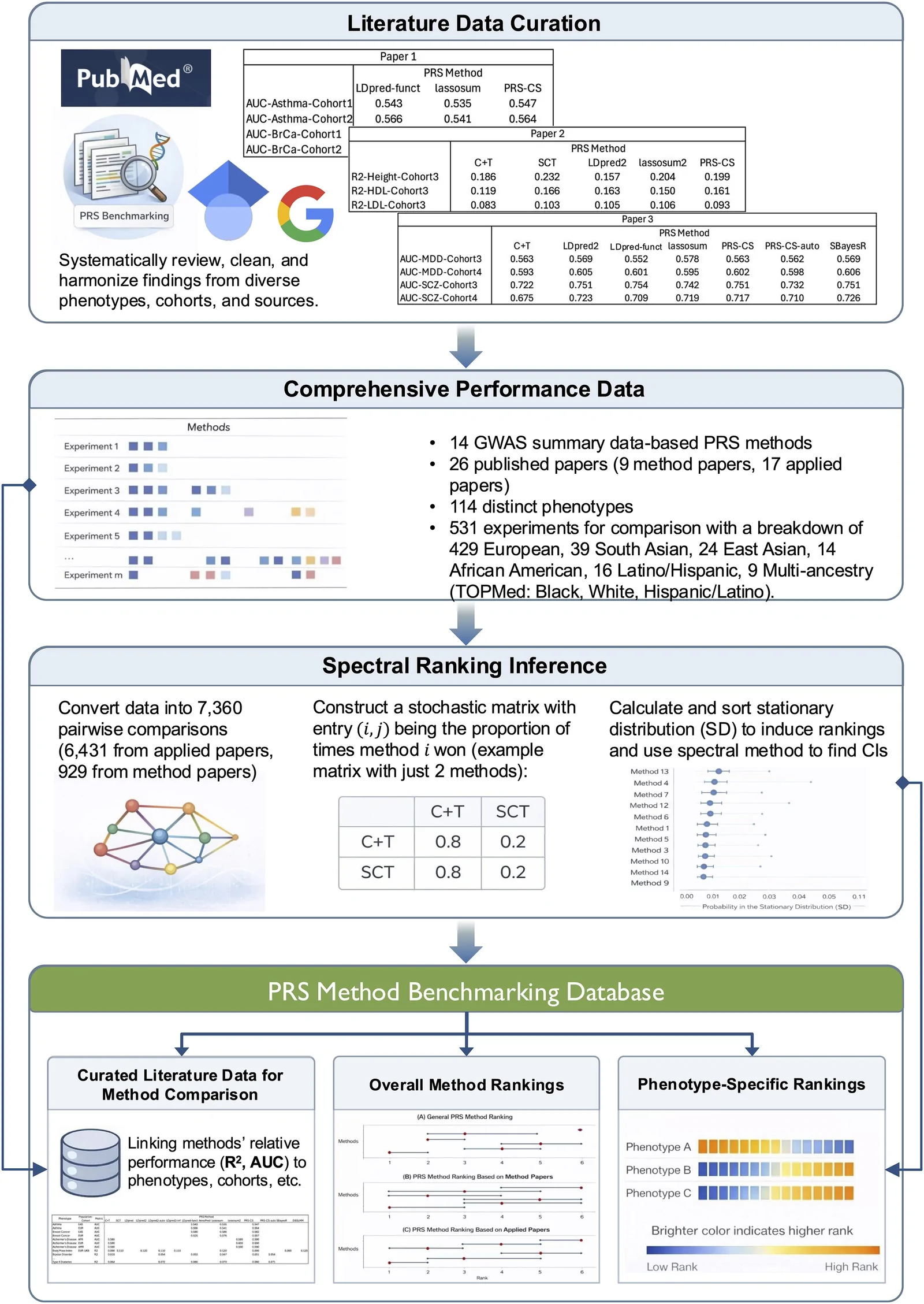
An overview of the study pipeline. The pipeline started from performing a review of the relevant literature, extracting and curating the relative performance data, to generating model ranks using spectral ranking inference. The final product is a PRS method benchmarking database consisting of three components: (1) curated literature data (*R*^2^ and AUC values) linking methods’ relative performance to specific phenotypes and cohorts; (2) overall method rankings based on all papers, the original method-development papers only, and the applied and benchmarking papers only; and (3) phenotype-specific rankings of the PRS methods.

The data collected came from two categories of paper sources, the first being the original method-development papers which introduced the PRS methods, and the second being the applied and benchmarking papers that studied the relative performance of multiple PRS methods on specific phenotypes or cohorts. Each method paper, besides the one that introduced the first method, C + T [12], had a data analysis section comparing the proposed method with the previously proposed methods or a subset of them on a set of phenotypes for illustrative purpose. These comparisons were mainly performed based on ${R}^2$ performance on predicting continuous phenotype values or AUC performance on classifying binary outcomes. We centralized these reported findings into a data table, with the rows corresponding to the various experiments on the same or different phenotypes and the columns corresponding to the PRS methods. The data collection was largely similar for the applied and benchmarking papers. Specifically, we conducted literature search iteratively over several weeks and completed along with data collection at the end of July 2025 (Supplementary Fig. S1). We searched for peer-reviewed publications on PubMed and search engines including Google and Google Scholar using combinations and variations of terms including “PRS method comparison,” “PRS method benchmarking,” and “real data analysis” (we do not consider papers that only report benchmarking results from simulation studies), together with the names of the selected PRS methods. A representative Boolean search string was (“polygenic risk score” OR PRS) AND (benchmarking OR “method comparison” OR “comparative evaluation”) AND (“real data” OR cohort OR biobank). We attempted to make our search as extensive as possible by continually performing our searches with variations in query construction over weeks. While there are additional papers that conducted PRS analysis on one phenotype as a small, ancillary part of a wider study, we did not gather such small-scale PRS performance data and instead focus on papers that proposed the methods analyzed or reported application and benchmarking of PRS methods as a part of their main results. Most papers were benchmarking efforts which had the comparison results structured in a similar way to the method papers: testing a series of methods against a series of phenotypes. Meanwhile, some other papers were testing the methods’ performance on just one phenotype. In these papers, the comparisons were done not by phenotype but by some other differentiating factors, such as study cohort. Thus, in these datasets, the columns still correspond to the methods being tested, but the rows now correspond to experiments/comparisons on the different cohorts, phenotypes, or studies. We centralized the results from these different datasets into a single table (Supplementary Table S1), which serves as the raw benchmarking data in our database and the basis on which our method rankings were constructed. Each row reports the comparison results from a specific method comparison experiment, and the columns report the 14 methods compared (each row will contain missing values on several method columns), phenotype, ancestry population of the validation set on which the performance was reported, evaluation metric used (e.g. ${R}^2$ or AUC), and the source publication.

In both types of papers, there were often real data applications accompanied by simulated studies. We include results only from real data applications but not simulation studies because first, we aim to rank the methods based on real-world performance, and second, simulation studies, which often generate synthetic data based on the assumed model assumptions, tend to favor the proposed methods. The collected data have a unique structure in that method comparisons are often conducted among subsets of methods on different phenotypes and cohorts, making the data table relatively sparse with missing entries and noncomparable rows. These unique data features cannot be easily handled by simple method benchmarking approaches such as taking an average across all applications. On the other hand, a statistically principled approach that can robustly integrate results across diverse studies can provide a better solution to our task.

### Spectral ranking inference

The spectral inference method, described in Fan et al. 2025 [24], recovers the global ranking of a set of items from a collection of incomplete pairwise comparisons by aggregating the observed comparison outcomes into a transition matrix corresponding to a random walk on the comparison graph. The stationary distribution of this matrix provides estimates of the latent preference scores, and the estimated scores are then ordered to obtain the items’ ranking. Specifically, the method begins by having a set of objects, numbered 1 through *n*, which we will call *Ω*. Additionally, there exists a collection of subsets of *Ω*, which we will call comparison sets. For each comparison set, there must exist a criterion for choosing the most favorable object from any given subset, which we will call the “winner” of the subset. Having this collection of subsets and their respective winners allows you to construct an *n× n* stochastic matrix representing the objects in *Ω*. Specifically, for nondiagonal entries, entry $\left(i,j\right)$ is an increasing function of the proportion of times the *i*th object in *Ω* is a winner of a comparison set in which the *j*th object is also a member. Each diagonal entry is defined as one minus the sum of the other (appropriately scaled) entries in its row, ensuring that every row sums to 1 and contains no negative elements. With the stochastic matrix constructed, one can then calculate the stationary distribution of the matrix by finding the top-left eigenvector of the stochastic matrix. The estimated ranking of the objects is now defined by sorting the objects in descending order of their probability in the stationary distribution. The intuition is that objects with larger stationary probabilities win more comparison sets and therefore should receive higher rankings [25, 26]. Through further calculations with the spectral method, we can derive uncertainty quantifications in the form of 95% CIs of the estimated rankings using the bootstrap method discussed in Fan et al. 2025 [24].

The spectral inference method is suitable for our problem setting for many reasons. First, it can aggregate sparse comparisons with many missing entries in the comparison table and provide uncertainty quantification for the resulted rankings. Second, the spectral method is used to rank preference scores over a general, heterogeneous choice sets, which is what our data provide as different method papers that benchmarked various subsets of our 14 PRS methods under heterogeneous study designs. Furthermore, the production of CIs allows for a richer analysis and comparison of methods within and between different rankings. In the context of our study, the set *Ω* corresponds to the 14 PRS methods considered. Different PRS benchmarking studies evaluated different subsets of these methods, resulting in an incomplete and heterogeneous comparison graph. To construct the comparison sets, we define the winner as the method achieving a higher predictive performance metric, such as prediction *R*^2^ or AUC, among methods evaluated within the same comparison using the same performance metric. We defined entry $\left(i,j\right)$ in the comparison table to be an increasing function of the proportion of times the *i*th object in *Ω* was a winner of a comparison set in which the *j*th object was also a member. In a comparison set with equal metric scores, no winner was declared and the data were not included. Hoping to avoid such situations and maximize the information obtained from the given data, when multiple methods were compared in the same experiment, we decomposed the multimethod comparison into pairwise comparison sets. For example, the comparisons among *k* methods were converted into $k\left(k-1\right)/2$ pairwise comparisons. For each comparison set, we defined the winning method as the method which had a higher prediction ${R}^2$ or AUC value. If two methods had the exact same reported performance metric, then no winner was declared, and that data point was excluded from the analysis (Fig. 1). This was not a common occurrence, though. As an example, consider an imaginary dataset containing the AUC performance of LDpred2, lassosum2, and PRS-CS for developing a PRS model for classifying the binary Type 2 Diabetes (T2D) status. Suppose there are three pairwise comparisons, with example AUC values in parathesis: [LDpred2 (.8) vs lassosum2 (.7)], [LDpred2 (.8) vs PRS-CS (.65)], and [lassosum2 (.7) vs PRS-CS (.65)]. The winner of each comparison, respectively, would be LDpred2, LDpred2, and lassosum2.

Because our construction converts continuous performance measures into binary win/loss outcomes, the stochastic matrix does not directly incorporate the magnitude of performance differences between methods: any nonzero difference in the performance metric results in the better-performing method being declared the winner. This may cause methods with very small performance differences to appear more distinguishable than they actually are. Therefore, we clarify that we do not imply that all retained differences in method performance are necessarily clinically or practically meaningful. To evaluate the robustness of the rankings to the threshold for meaningful differences, we performed a near-tie sensitivity analysis, as described in Results section, which showed the robustness of the inferred rankings to alternative definitions of a near tie. In addition, comparisons derived from the same study or partially overlapping datasets may not be fully independent, which may affect uncertainty quantification. Therefore, the resulting CIs should be interpreted as reflecting uncertainty under the constructed comparison framework.

The spectral ranking framework also relies on sufficient connectivity of the comparison graph. Specifically, methods with limited comparison evidence or methods that are disconnected from the rest of the comparison graph may not have reliably estimated rankings. Furthermore, because PRS performance can vary substantially across phenotypes, cohorts, ancestries, GWAS sample sizes, and other data characteristics, the resulting ranking should be interpreted as an aggregate summary of performance across the available literature rather than as a universally optimal ordering of PRS methods.

## Results

### Curated raw method comparison data

The curated raw benchmarking data in the form of predictive performance metrics AUC and *R*^2^ in Supplementary Table S1 record the relative performance within subsets of the 14 PRS methods in various contexts. We observe a large variation in the pairwise differences in method performance (Supplementary Fig. S2); while the difference in performance between the rank-1 and rank-2 methods are smaller than average pairwise difference, they are far larger than a near-tie difference (e.g. an absolute difference below 0.01), suggesting that identifying a top-performing method does matter in many applications (Supplementary Fig. S3). Furthermore, within-method performance varies greatly across studies/comparisons (Supplementary Fig. S4), partially due to the varying study design (e.g. covariates adjusted, study cohort, targeted phenotype). This suggests that naive approaches cannot appropriately summarize the raw comparison data and thus there is a need for a robust ranking analysis to complete our benchmarking database.

### The general ranking of methods

We constructed and compared three sets of rankings based on method performance reported in the literature. Detailed information on the extracted literature data is summarized in Supplementary Table S1. We first constructed the overall rankings by combing data from both the original method papers and the applied/benchmarking papers. Figure 2A presents the overall ranking results: the methods were listed chronologically based on the publication date of their method papers, with the very first (C + T) being at the top of the method axis and the newest (lassosum2) being at the bottom. LDpred2 and AnnoPred are ranked the highest with tight CIs that showed significant superiority over most of the other methods except lassosum2, SCT, and LDpred-funct. C + T, LDpred, and LDpred2-inf are ranked the lowest with similarly high degrees of confidence that clearly distinguish them from higher ranked methods. The other nine methods tend to cluster in the middle of the ranking and have wider CIs, suggesting a similar performance overall. A large number of comparisons conducted for a method did not necessarily lead to a narrow CI for the method’s rank. For example, SBayesR had the third largest number of comparisons but also had a rank with a second widest CI (Fig. 2A). The occurrence of the wide, overlapping CIs of rankings across most methods may be due to the large variation in genetic architectures across different phenotypes (e.g. heritability, effect size distribution of genetic variants) and data settings (e.g. GWAS sample size, ancestral population) that could lead to highly heterogeneous relative performance between different methods.

**Figure 2:**
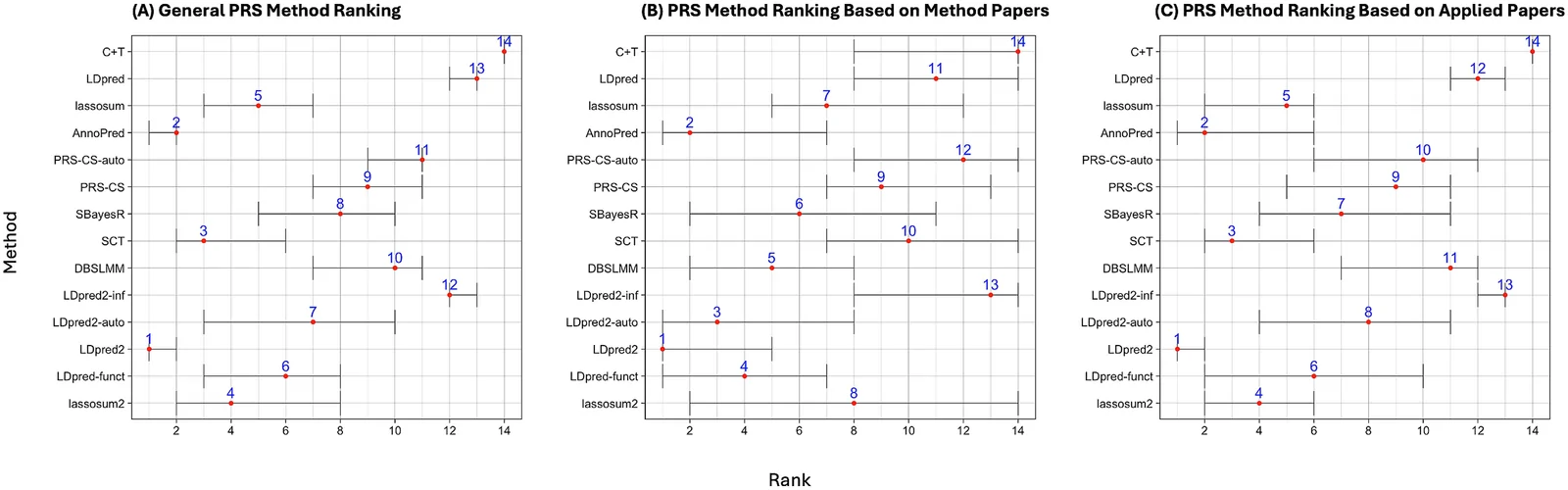
Spectral method inferred rankings of 14 PRS methods based on benchmarking results reported in the literature. (A) General method rankings estimated using data from all papers. (B) Method rankings estimated based on data from the original method-development papers. (C) Method rankings estimated based on data from the subsequent application and benchmarking papers. The dots and bars show point estimates and 95% CIs, respectively, of the rankings. The methods are ordered chronologically (by publication date of their method papers) with C + T being the first method and lassosum2 being the latest. Detailed method performance data and pairwise win/loss statistics supporting these rankings are provided in Supplementary Tables S1–S3.

We further generated two separate rankings based on results reported in the original method papers and in the applied/benchmarking papers, respectively. Detailed method comparison results, including the number of wins and losses of each method against others, are reported in Supplementary Table S2. Figure 2B shows the ranking produced based only on results collected from the method papers. Given that the experiments conducted in the method papers were typically for illustrative purpose, comparisons were conducted only on a limited number of phenotypes and cohorts, which could partially explain the wider CIs of the rankings compared to those in Fig. 2A. Even so, we can observe approximately two clusters of methods where one (including LDpred2, AnnoPred, LDpred2-auto, LDpred-funct, and DBSLMM) outperforms the other (including PRS-CS, SCT, LDpred, PRS-CS-auto, LDpred2-inf, and C + T). Three other methods, SBayesR, lassosum, and lassosum2, show rankings with wider CIs that overlap with both clusters. A notable pattern is that the rankings tend to be higher for methods proposed more recently: most of the lower-ranking methods are those proposed earlier (top half of the method axis, C + T through SBayesR), while most of the higher-ranking methods are generally those proposed later (bottom half, SCT through lassosum2). Such a general trend is consistent with the claim in the method papers that newer methods perform better, with a few exceptions: AnnoPred ranking second, LDpred2-inf ranking 13th, and the newest method lassosum2 ranking 8th, but with wide CIs that overlap with that of many other methods.

As a comparison, we further conducted method ranking only using data from application and benchmarking papers (Fig. 2C). This serves as a control group with objective method benchmarking on a wider range of phenotypes. Detailed method comparison results, including the number of wins and losses of each method against others, are reported in Supplementary Table S3. Given that these applied papers report a much larger number of comparisons (6431 compared to 929 from method papers), the CIs are generally tighter, allowing for more statistically significant differentiations to be made. This leads to some similar (as compared to rankings based on method papers in Fig. 2B) but more significant rankings. For example, LDpred2-inf, LDpred, and C + T, which are ranked among the worst methods based on method papers, are now still ranked among the worst methods, but with higher certainty. LDpred2 and AnnoPred remain the top two methods with higher certainty. Among the remaining methods in the middle, some have similar rankings as before but with narrower CIs, such as SBayesR (6 to 8), lassosum (7 to 5), PRS-CS (9 to 10), and PRS-CS-auto (12 to 9). However, three methods have a significant change in ranking positions: SCT goes up from 10th to 3rd and DBSLMM goes down from 5th to 11th (both having narrower CIs), while lassosum2 goes up from 8th with a wide CI to 4th with a much narrower CI. These wide swings in performance are likely due, in part, to the greater variability in phenotypes present in the applied paper dataset than in the method paper dataset. The method papers typically tested the same few phenotypes (e.g. cancers and blood lipid traits) and might not provide a holistic evaluation of a method’s general performance. From the applied paper-based ranking, some of the earliest proposed methods, such as lassosum, AnnoPred, and SCT, are ranked in the top half of methods with high confidence, while DBSLMM, a more recently proposed method, is ranked in the lower half of methods. These results suggest that, with a larger number of applications across a wider variety of phenotypes, the overall performance of PRS methods seem to be less related to their publication date (compared to the findings from method paper-based ranking in Fig. 3B). Despite some apparent differences, the two rankings in Fig. 2B and C do assign the same methods to be ranked first and second, as well as the same methods ranked last and second to last. Note that, while LDpred2-inf and LDpred2-auto were listed among the recently proposed methods, they were proposed along with LDpred2 as a simple version and a tuning-parameter-free version, respectively, of LDpred2. LDpred2-inf was expected to have suboptimal performance, which may explain its low rank in both rankings.

**Figure 3:**
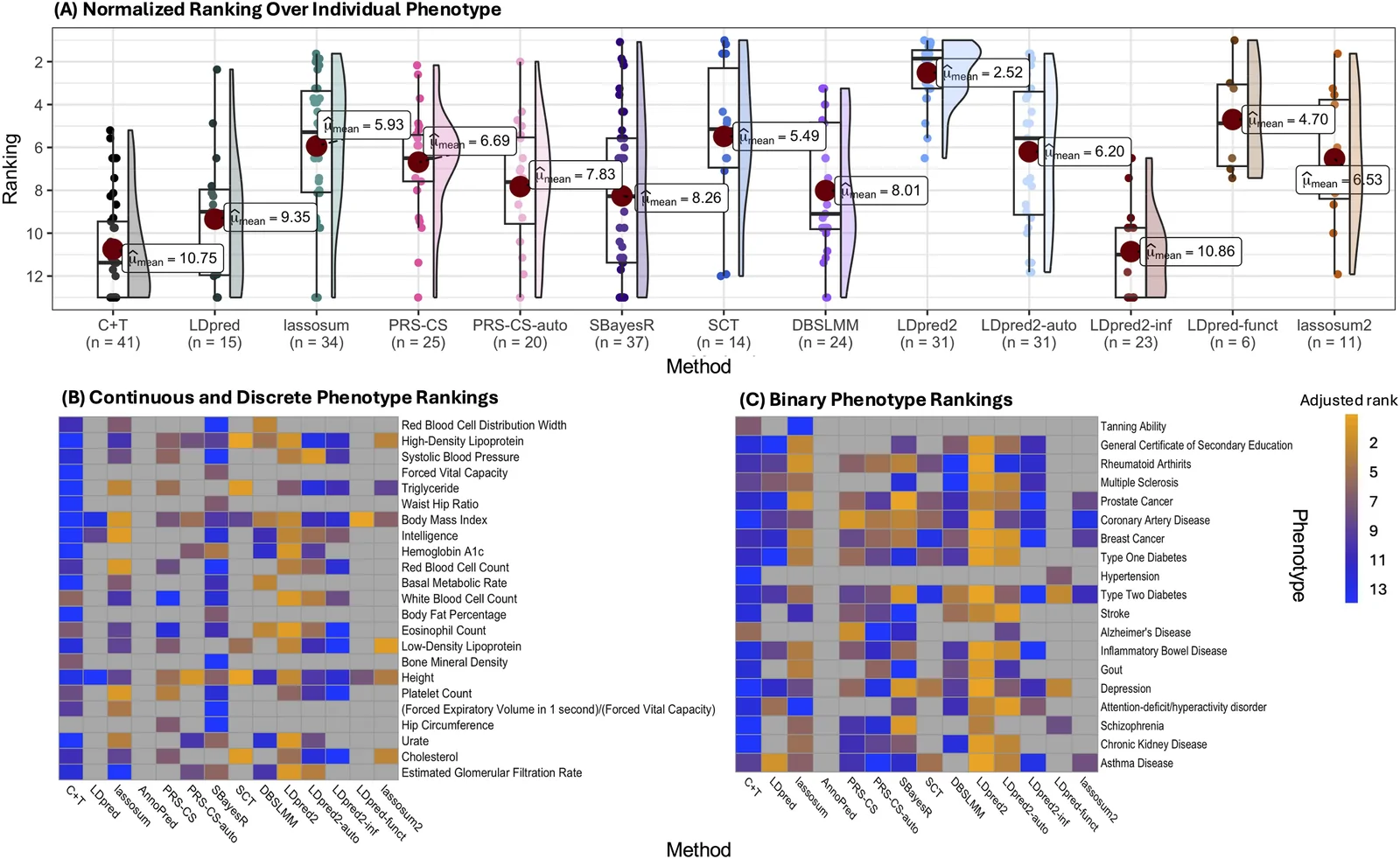
Phenotype-specific rankings of PRS methods. (A) Half violin plot showing normalized ranking across eligible phenotypes. The dots in dark red show the average inferred rankings of each method across all eligible phenotypes. The expression (*n* = *x*) under each method shows the number of phenotypes the method passed the filtering check and was ranked for. (B) A heatmap showing the ranks the methods achieved when tested against given continuous and discrete phenotypes. (C) A heatmap showing the ranks the methods achieved when tested against given binary phenotypes. The color ranges from orange, representing high ranks, to blue, representing low ranks. Gray cells occur when the method was not ranked on the specific phenotype because it was removed during the described filtering process. AnnoPred failed to be compared at least 10 times for any phenotype and was thus omitted from the phenotype-specific comparison. Details of the normalized ranks for continuous and binary phenotypes are summarized in supplementary tables 5 and 6, respectively.

Overall, the two sets of rankings based on separate data sources from method papers and applied papers provide complementary observations of PRS method performance. The highest and lowest ranked methods are relatively consistent between the two rankings but with the applied rankings providing narrower CIs: possibly due to the much larger number of experiments included. For the majority of the methods, the CIs of their rankings are wide, showing no significant difference across most of the method pairs. Nonetheless, there does exist statistically significant difference in overall method performance, especially within the rankings based on results reported in applied papers. These rankings, along with the raw win and loss numbers reported in Supplementary Tables S2 and S3, provide researchers with unique perspectives about PRS method performance with diverse benchmarking summary of the PRS method performance based on different literature sources, which could help better inform the choice of PRS methods for future PRS construction.

### Phenotype-specific analysis

The rankings reported in Fig. 2 were calculated by aggregating benchmarking results from the literature across a total of 114 distinct phenotypes. These rankings provide an overall assessment of the relative performance of PRS methods: reflecting their robustness and general applicability across diverse genetic architectures and application settings. However, the relative performance of PRS methods is known to be phenotype dependent. Thus, we further investigate phenotype-specific rankings as a complementary analysis, which could provide insights into method performance within individual trait categories and inform method selection for future PRS model development targeting on specific phenotypes.

To construct phenotype-specific rankings, a naive approach is to partition the dataset into phenotype-specific sub-datasets and calculate ranking on each sub-dataset. This approach, however, presents a unique challenge, as some methods had very few comparisons and thus their rankings were uninformative or simply misleading. This is because there were many phenotypes for which one method had >5 comparisons but always won. This situation led to a stationary distribution that put all its mass on the method which had a 100%-win rate and 0% on all other methods. This created two problems: (1) the other 13 methods could not be ranked, and (2) it is unconvincing that this method ranked first when it was only compared 5 times. To address this issue, we filtered out any method which had fewer than 10 comparisons for a given phenotype (see the Phenotype Filtering Threshold Analysis subsection for a discussion on the selection of 10 as the threshold). This step ensured that all the methods present after filtering had enough comparisons for a given phenotype to produce a meaningful ranking. During the process, AnnoPred failed to be compared at least 10 times for any phenotype and was thus omitted from the phenotype-specific comparison. This approach largely solved the stated issue. However, there were still a few instances where a method won all its comparisons. Given that the method had over 10 comparisons, though, there was enough data to believe this wasn’t an anomaly. However, the 0%-win rate assigned to other methods would make the ranking impossible for all other methods. To ensure all methods could be ranked, we thus removed this top method from the sub-dataset, labeled it as the top ranked method, and then reran the ranking calculation on the remaining methods. This had to be an iterative process, because once you removed the highest ranked method from the dataset, the second highest ranked method could have been undefeated amongst all remaining methods. Thus, we iterated this process until a final ranking across all methods was achieved.

After method filtering, each phenotype *p* had a ranking of a subset of the 13 methods (AnnoPred was filtered out due to insufficient comparisons), which we will refer to as ${M}_p$. The cardinality of ${M}_p$ varies from 0 to 13 depending on *p* because each method had a variable number of comparisons for any given phenotype *p*. The varying cardinality makes it difficult to compare rankings across phenotypes because a ranking of first out of two methods should hold less weight than a ranking of first out of 13 methods. Due to this substantially varying number of PRS methods evaluated across different phenotypes, raw phenotype-specific ranks are not directly comparable across traits. We therefore applied a coverage-aware rank adjustment that rescales phenotype-specific ranks to a common 1–13 scale while penalizing favorable ranks obtained from phenotypes with fewer eligible methods. This adjustment intentionally penalizes favorable ranks obtained from sparsely compared phenotypes while preserving the relative ordering of methods within each phenotype, thereby reducing the risk of over-interpreting apparently high ranks that are supported by only a small comparison set. The coverage-aware rank adjustment on ranking across methods for each phenotype works as follows. If a method achieved a rank *r*, we defined its final ranking ${r}_f$ as


$$ {r}_f=r\cdotp \frac{13}{\left|{M}_p\right|} $$

This normalizing factor $13/\left|{M}_p\right|$ penalizes rankings which had a small number of methods present. The more methods present, the smaller the normalizing factor is. At its smallest, the normalizing factor is 1 and has no impact on the ranking, allowing the best method to achieve a final ranking of 1. At its largest, the normalizing factor is 6.5 (when ${M}_p$ contains the smallest possible number of methods, 2), making the best method’s final ranking 6.5. This ensures that methods are only listed as ranking highly if there is an adequate amount of data to support that conclusion. Information on the number of comparisons by phenotype is summarized in Supplementary Table S4. The final rankings after coverage-aware rank adjustment across 23 continuous and 19 binary phenotypes for which PRS methods were adequately compared are summarized in Fig. 3.

We observe that the average method performance across phenotype in Fig. 3A yields a very similar ranking to the overall ranking in Fig. 2A. As expected, the large variation in most methods’ ranks across phenotypes suggests that the generally “worst” method could outperform the generally “best” method when looking at a specific phenotype. For example, C + T was ranked with high certainty as the worst method across the applied papers, but when looking at Fig. 3C one can find it is ranked 2nd for Alzheimer’s disease. While LDPred2, which was ranked with high certainty as a top 2 method, was ranked 13th for Platelet Count (Fig. 3B, Supplementary Table S5). These phenotype-specific rankings presented in Fig. 3B and C clearly articulate the robustness and specialty (on phenotypes highlighted with light orange) of each PRS method (see Supplementary Tables S5 and S6 for detailed ranks). For example, we can observe a consistent superiority and high robustness of LDpred2 across most phenotypes considered, while SBayesR appeared to be a suitable choice for training PRS models for Schizophrenia and Depression, both being highly polygenic phenotypes that have widespread causal genetic variants across the genome. In general, these phenotype-specific rankings, alongside the overall rankings, synthesizing benchmarking efforts from the literature, provide a comprehensive landscape of PRS method comparison that could inform future PRS training. Researchers can refer to the relevant part of the phenotype-specific rankings (Fig. 3, Supplementary Tables S4–S6), along with the raw benchmarking data, we extracted from the literature reporting detailed method comparison results by phenotype, population, and cohort (Supplementary Table S1), for choosing PRS methods that could be most promising for specific phenotypes of interest.

### Near-tie sensitivity analysis

Our rankings are derived from the stationary distribution of a stochastic matrix. Entries in the stochastic matrix are derived from an increasing function of the number of times a method performs better than the other methods it is compared to. The main results presented in Figs 2 and 3 declare a method *X* to have performed better than a method *Y* so long as *X* presents a better metric (e.g. AUC) than *Y* in a comparison. Such winner-based ranking ignores effect sizes and may over-emphasize near-tie differences, such as 0.001, which lack practical or statistical significance. To evaluate the robustness of our ranking approach to the threshold for performance difference, $\Delta \mathrm{AUC},$ or $\Delta{R}^2$, we performed a near-tie sensitivity analysis, where we did not include results from methods whose metrics were within a certain threshold. As shown in Fig. 4, method rankings remained overall similar with a threshold for performance difference from 0 to 0.01. The largest change in point estimate was within two ranking positions, and all changed rankings remained within the CIs of the original ranks. Furthermore, CIs remained of similar size until the threshold reached above the point of indistinguishable difference. These results suggest that virtually indistinguishable differences did not play a large role in determining the rankings reported. Nevertheless, we would like to clarify that the estimated rankings summarize relative ordering of the methods and should be interpreted alongside the curated raw performance metrics (e.g. prediction *R*^2^ and AUC values) in Supplementary Table S1, which is a part of our literature-derived PRS method benchmarking database.

**Figure 4:**
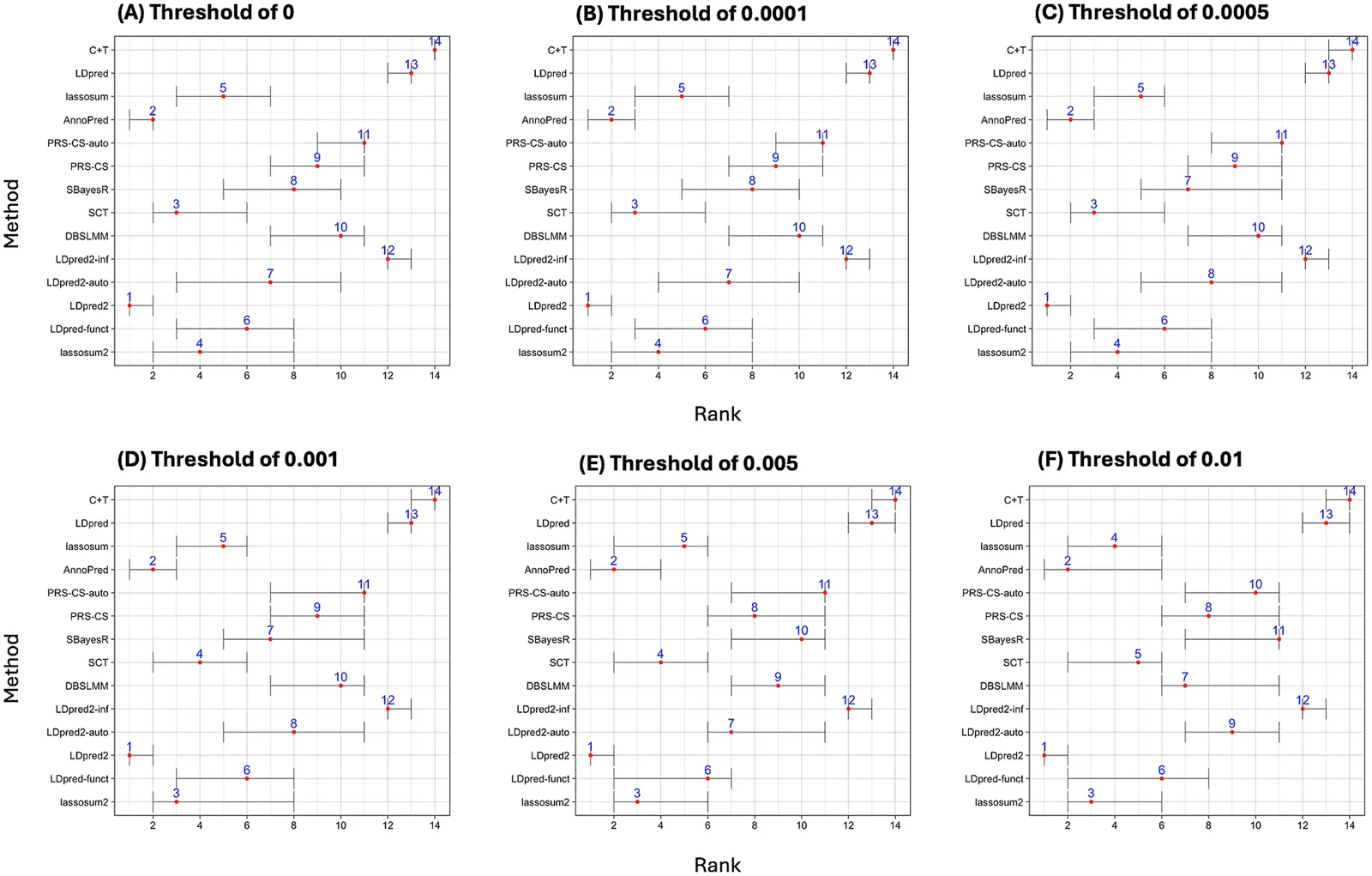
Estimated overall rankings with varying inclusion thresholds for absolute performance differences in AUC or *R*^2^ on phenotype prediction.

### Phenotype filtering threshold analysis

For the phenotype-specific analyses, we excluded a method for a given phenotype if it had fewer than 10 pairwise comparisons. This filtering step was intended to reduce instability in rankings based on limited evidence, particularly situations in which a method could appear to rank first solely because it won all of a small number of comparisons. Because the choice of 10 was not theoretically determined, we evaluated the robustness of the phenotype-specific rankings across filtering thresholds ranging from 5 to 20 in increments of 5. Thresholds of 10 and 15 yielded broadly similar ranking patterns, whereas a threshold of 5 admitted many sparsely supported estimates and produced substantially greater variability (Fig. 5). In contrast, thresholds of 20 or higher resulted in a notable reduction in the number of methods and phenotypes retained for analysis, thus limiting the breadth and interpretability of the comparison. We therefore retained a threshold of 10 as a pragmatic balance between ranking stability and data coverage.

**Figure 5:**
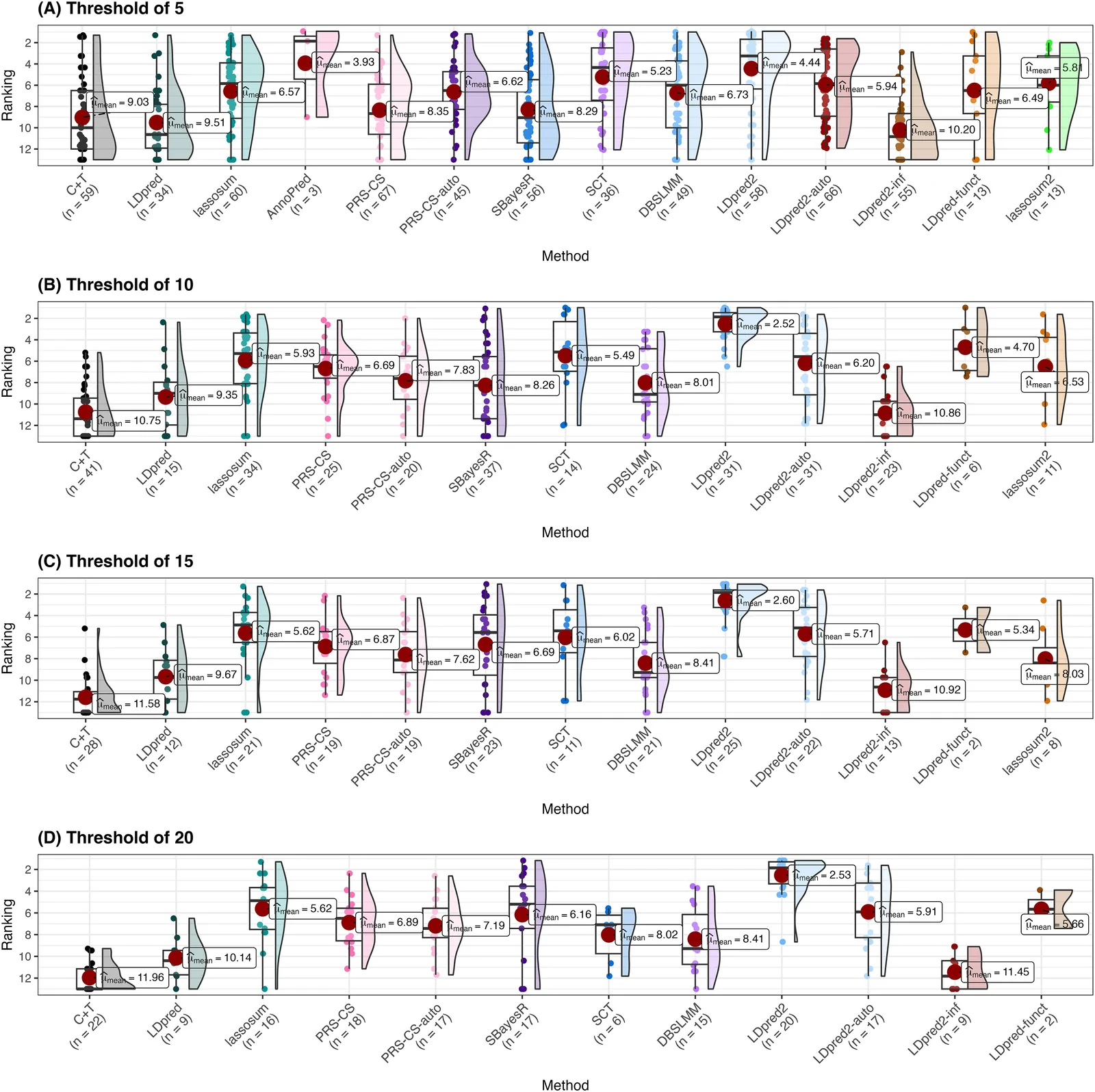
Phenotype specific rankings with varying minimum number of comparisons on each phenotype. Each graph shows the normalized phenotype specific ranking for a different threshold value. The threshold value is the minimum number of times a method must have been compared against a phenotype for it to be included in that phenotype’s phenotype specific ranking.

These results illustrate the expected tradeoff between evidential support and breadth of coverage. Increasing the threshold for the minimum number of comparisons required strengthens the empirical support for phenotype-specific ranking, but at the same time excludes more methods and phenotypes from the analysis. Conversely, lowering the threshold increases coverage at the cost of greater instability in sparsely supported rankings. Given the current amount of benchmarking evidence available in the literature, a threshold of 10 provides a reasonable balance between these considerations. As additional benchmarking data accumulate, future updates of the database may support the use of more stringent thresholds without substantially reducing the number of methods and phenotypes that can be ranked. In our algorithm, we allow users to customize this threshold to allow customized ranking analysis (https://github.com/codeitchris/PRS-Ranking-Paper-Code-and-Data).

## Discussion

Understanding the relative performance of computational methods through synthesis of the existing literature can yield important insights into their real-world utility and inform future methodological development and implementation. In the context of PRS modeling, new methods continue to emerge, while comparative evaluations are typically conducted on limited subsets of methods across disparate studies. As a result, the available benchmarking evidence remains fragmented and inconsistently reported across limited studies, making it difficult to identify the optimal methods for specific applications. Establishing a comprehensive benchmarking resource that integrates varying sources of available evidence from the literature is therefore essential for thoroughly investigating the relative performance of PRS methods and guiding their future applications. Our study leads such an effort by establishing a PRS method benchmarking database, summarizing the relative performance of PRS construction methods with available benchmarking data from the literature. We curated publicly available benchmarking results for 14 GWAS summary data-based PRS construction methods from 26 published peer-reviewed publications and applied a statistically rigorous ranking inference approach with uncertainty quantification to integrate these sparse and heterogeneous comparison results. We derived PRS methods’ rankings for their predictive performance based on (1) original method-development papers only, (2) applied and benchmarking papers only, and (3) both original method-development papers and applied and benchmarking papers. Although the current data size (7360 pairwise comparisons in total, Supplementary Tables S1–S3) yielded relatively wide CIs for the ranks of many methods, several methods consistently clustered near the top (LDpred2 and AnnoPred) or the bottom (C + T and LDpred2-inf) across different rankings. Finally, we ranked methods’ performance by individual phenotypes, revealing more detailed and highly heterogeneous rankings across different categories of phenotypes. No clear trend of method ranks by publication date was observed, suggesting that the newer methods do not necessarily outperform earlier ones. However, the large variability in ranks and failure to differentiate ranks among most of the methods suggest that there are no “best” methods, and that future method development could continue being specialized for specific genetic architectures or introducing more flexibility in handling the genetic nuances across phenotypes.

Given current data availability, our PRS method ranking is restricted to 14 single-ancestry methods that construct PRS using a single GWAS summary-level dataset, while other recently proposed methods in this category were not considered due to insufficient benchmarking data to support reliable ranking. Beyond this setting, a growing body of work has focused on extending standard PRS modeling frameworks to improve predictive performance and generalizability, such as multi-ancestry methods [27–30], multi-trait methods [31], and methods incorporating admixed or local ancestry information [32–34]. These methods were also excluded from the current version of our benchmarking database due to limited benchmarking evidence. We note, however, that we interpret our benchmarking database, including the curated literature evidence and method ranking analysis framework, as a dynamic integration of the currently available benchmarking literature, rather than as a comprehensive assessment of all existing PRS methods. A comprehensive understanding of all methods will not be possible without future large-scale benchmarking efforts on the many currently under-assessed methods. We are thus advocating future large-scale PRS method benchmarking studies with our literature-based benchmarking effort in this study. As additional benchmarking studies become available, our database can be naturally expanded to incorporate more methods and new evidence for existing methods utilizing our ranking framework, allowing the database to evolve with the PRS literature [35–38].

The constructed PRS method rankings should be interpreted with caution. First, the rankings reported in this study serves as an integration of the currently available benchmarking literature rather than as a definitive assessment of all existing PRS methods. While our analyses have synthesized benchmarking results from the literature, the phenotypes covered are still limited in number, suggesting that only a partial insight is available from our current ranking database. Nevertheless, we anticipate that a more comprehensive analysis will be feasible in the near future, given recent large-scale benchmarking efforts reported in preprints that evaluated relative performance of PRS methods across a broader range of phenotypes [39, 40]. Second, our ranking framework provides a robust way to summarize results across different studies and phenotypes, for which the predictive performance metrics such as *R*^2^ or AUC are not directly comparable, and the comparison tables are often sparse. While the spectral inference approach allows a robust ranking under these conditions, it characterizes relative ordering in rather than the magnitude of performance differences in specific metrics. Consequently, such rankings, especially the phenotype-specific rankings, should be interpreted in conjunction with the corresponding *R*^2^ or AUC values in our collected literature database to more fully inform future method implementation.

Several additional limitations concern the statistical evidence underlying the rankings. All comparisons are currently weighted equally across studies, although studies may differ in sample size and in the uncertainty of their reported *R*^2^ or AUC estimates. We currently do not take into account study-specific uncertainty because such information was unavailable for most studies. As more complete uncertainty estimates become available, the framework could be extended to incorporate study-specific weights, such as inverse-variance weights, to further refine the rank inference. In addition, comparisons across studies may not be fully independent because multiple studies may use the same or partially overlapping GWAS summary statistics or validation cohorts. In our curated database, clearly overlapping benchmarking settings were uncommon, but comprehensive participant-level overlap could not always be determined from the published information. Our current spectral ranking procedure does not explicitly model such cross-study dependence; consequently, residual correlation among comparisons could lead to some under-estimation of uncertainty, even if its impact on the estimated rankings is limited. Future extensions could incorporate study- or dataset-level clustered resampling or other dependence-aware inference procedures when sufficiently detailed information on sample overlap is available. Additionally, we adopted pairwise decomposition of multiway comparisons within each study because it provides a common representation for integrating heterogeneous benchmarking results across studies that evaluate different subsets of PRS methods while retaining the observed relative-ordering information within each experiment. While converting multiway comparisons into pairwise comparisons may introduce within-study correlations, the converted pairwise comparisons should be valid to use, as a previous paper by Han and Xu has noted [41]. Although our sensitivity analysis indicated that decomposing multiway comparisons into pairwise comparisons had little impact on the estimated point rankings, this dependence is not explicitly incorporated into the current uncertainty quantification and may therefore lead to underestimation of ranking uncertainty (Supplementary Fig. S7). Finally, our current rankings focus exclusively on predictive accuracy and do not consider computational efficiency or computational resource requirements, which are also important practical considerations for PRS method selection. Finally, our analyses primarily focused on complex human phenotypes, while other phenotypes classes, such as molecular traits and imaging traits, that also benefit from PRS modeling [42–44], could be examined as well [42].

In summary, our PRS method benchmarking database, including the curated literature data on methods’ relative performance (Supplementary Table S1) and generated method rankings (Figs 2 and 3), provide a valuable reference resource for PRS method comparison and implementation across a broad range of human traits and diseases. We further identify a series of directions for extending this resource, highlighting the need for future efforts to continuingly update the constructed database as new methods and benchmarking studies emerge. Such efforts will enable the development of a dynamic and practical reference framework to guide PRS method selection and implementation in future research and applications. More broadly, we hope that the PRS application of our general analysis framework for curating and synthesizing literature evidence to construct benchmarking databases for computational methods could provide insights into the feasibility of extending the framework to other domains where comprehensive benchmarking of computational methods is needed.

**Key Points**

- Benchmarking evidence for polygenic risk score (PRS) methods is fragmented across method-development and applied studies. Each compares a different subset of methods on different phenotypes and cohorts, making it’s hard to evaluate relative comparison of methods.
- We curated 7360 pairwise comparisons from 531 real-data experiments in 26 published studies covering 114 phenotypes. We then used spectral ranking inference with confidence intervals (CIs) to rank 14 single-ancestry, genome-wide association studies summary statistics-based PRS construction methods.
- LDpred2 and AnnoPred ranked highest and C + T ranked lowest based on rankings derived from method-development papers only, applied/benchmarking papers only, or the combined evidence. Most other methods showed ranks with overlapping CIs, suggesting broadly similar overall performance.
- Phenotype-specific rankings revealed substantial heterogeneity in method performance across 42 phenotypes with sufficient data to support phenotype-specific analysis, while also highlighting the level of robustness and phenotype-specific strengths of individual methods.
- The curated literature data on method performance, derived rankings, and open-source pipeline for performing customized ranking analysis form a literature-based PRS method benchmarking database that can be continuously updated with emerging new methods and published benchmarking results to guide future PRS method selection and implementation.

## Supplementary Material

Supplementary_Materials_bbag598

## Data Availability

All code used for data cleaning, graph making, ranking calculation, and the collected datasets on method performance are available at https://github.com/codeitchris/PRS-Ranking-Paper-Code-and-Data.
