# Supplementary material for: A literature-derived benchmarking database for single-ancestry polygenic risk score construction methods with spectral ranking inferences
Source: Brief Bioinform. 2026 Oct 30;27(5):bbag598. doi: 10.1093/bib/bbag598 (PMC13720734; doi:10.1093/bib/bbag598)
Supplement: Supplementary file 1 [file supplementary_materials_bbag598.zip › Supplementary_Materials_bbag598/Supplementary_Materials_09.20_bbag598.docx]

**Supplementary Materials for**

**A Literature-Derived Benchmarking Database for Single-Ancestry Polygenic Risk Score Construction Methods with Spectral Ranking Inferences**

**This file includes:**

Supplementary Figures 1 to 8

**Other supplementary materials for this manuscript include:**

Supplementary Tables 1 to 6 (.xlsx)

**Supplementary Figure 1: workflow of the literature review, paper inclusion, and data curation procedure.** The final curated dataset contains 26 peer-reviewed publications, including 9 method papers and 17 applied/benchmarking papers, 114 phenotypes, 531 experiments for comparison (441 European, 17 African American, 10 Hispanic/Latino, 21 East Asian, 39 South Asian, and 3 Asian *unspecified*), with a total of 7,360 pairwise comparisons.

**Supplementary Figure 2: Comparison of the prediction performance of the best-performing and worst-performing methods for (A) binary outcomes (AUC) and (B) continuous outcomes (R2) across comparisons.** The dashed red lines represent threshold of .005 for the absolute performance difference. Detailed method performance data are provided in Supplementary Table 1.

**Supplementary Figure 3: Comparison of the prediction performance of the top two performing methods for (A) binary outcomes (AUC) and (B) continuous outcomes (R^2^) across comparisons.** The dashed red lines represent threshold of .005 for the absolute performance difference. Detailed method performance data are provided in Supplementary Table 1.

**Supplementary Figure 4**: **Raincloud plots of each method’s raw performance data for (A) binary outcomes (AUC) and (B) continuous outcomes (R^2^) across comparisons.** The methods are ordered chronologically (by publication date of their method papers). Detailed method performance data are provided in Supplementary Table 1.

**Supplementary Figure 5: Histogram of pairwise absolute difference in (A) AUC and (B) prediction R^2^ over all method pairs across comparisons.** The dashed lines annotate different thresholds for the absolute difference.

**Supplementary Figure 6: Histogram of pairwise absolute difference in (A) AUC and (B) prediction R^2^ between rank-1 and rank-2 methods in each comparison.** The histogram shows the distribution of the absolute differences between the top two performing methods from each comparison. The annotations provide exact figures on values of interest.

**Supplementary Figure 7: Estimated PRS method rankings based on pairwise comparison data (left panel) versus the full multi-way comparison data (right panel).**
